# Crystal structure of 2-(2,5-di­meth­oxy­phen­yl)benzo[*d*]thia­zole

**DOI:** 10.1107/S2056989022003279

**Published:** 2022-03-31

**Authors:** Nadia H. Metwally, Galal H. Elgemeie, Peter G. Jones

**Affiliations:** a Chemistry Department, Faculty of Science, Cairo University, Giza, Egypt; b Chemistry Department, Faculty of Science, Helwan University, Cairo, Egypt; c Institut für Anorganische und Analytische Chemie, Technische Universität Braunschweig, Hagenring 30, D-38106 Braunschweig, Germany

**Keywords:** benzo­thia­zole, pepsin catalysis, crystal structure

## Abstract

The title compound, C_15_H_13_NO_2_S, was synthesized efficiently in the solid state by exploiting pepsin catalysis. The ring systems are nearly coplanar [inter­planar angle of 5.38 (2)°] with an associated intra­molecular S⋯O=C short contact of 2.7082 (4) Å. The packing involves C—H⋯O, C—H⋯π and π–π contacts.

## Abstract 2

The title compound was synthesized efficiently in the solid state by exploiting pepsin catalysis. The ring systems are nearly coplanar. A short intra­molecular S⋯O=C contact is observed.

## Chemical context

Although countless synthetic methods are widely available, new and more efficient procedures or approaches are always needed. Enzymes, as ‘green’ catalysts for modern organic synthesis, have attracted increased attention because they may provide alternative and sustainable processes, thus helping to minimize the release of haza­rdous substances into the environment (Witayakran & Ragauskas, 2009 ▸). Pepsin, a kind of hydro­lase, belongs to the family of aspartic acid proteases and is involved in chemical digestion of protein (Cooper *et al.*, 1990 ▸; Lin *et al.*, 1989 ▸). Pepsin-catalysed aldol (and other) reactions have been developed (Li *et al.*, 2010 ▸; He *et al.*, 2016 ▸; Zongbo *et al.*, 2017 ▸).

2-Aryl-benzo­thia­zoles are a class of nitro­gen-containing heterocyclic compounds that can be found in a variety of natural and synthetic compounds. In view of their biological and pharmacological characteristics, we are inter­ested in developing synthetic strategies for heterocyclic ring systems containing a benzo­thia­zole moiety; these have shown significant biological activity as novel anti­viral and anti­microbial agents. (Azzam *et al.* 2017*a* ▸,*b* ▸, 2020*a* ▸,*b* ▸,*c* ▸, 2021 ▸; Elgemeie *et al.*, 2000*a* ▸,*b* ▸, 2020 ▸). The conventional synthesis of 2-aryl-benzo­thia­zoles, which involves heating a mixture containing 2-amino­thio­phenol (**1**), is disadvantageous because **1** is extremely unstable in air and highly toxic. In a continuation of our recent research in developing ‘green’ and simple syntheses of novel heterocyclic compounds (Metwally *et al.*, 2020 ▸, 2021*a* ▸,*b* ▸), we have now synthesized 2-(2,5-di­meth­oxy­phen­yl)benzo[*d*]thia­zole (**3**) using pepsin as the ‘green’ catalytic reaction. Thus, a mixture of **1** and 2,5-di­meth­oxy­benzaldehyde **2** was ground in a mortar with 0.05 g pepsin for 10 minutes, providing the desired product **3** in 97% yield. The nature of compound **3** was confirmed by spectroscopic analysis and by the single-crystal X-ray structure reported here.

## Structural commentary

**Figure 1:**
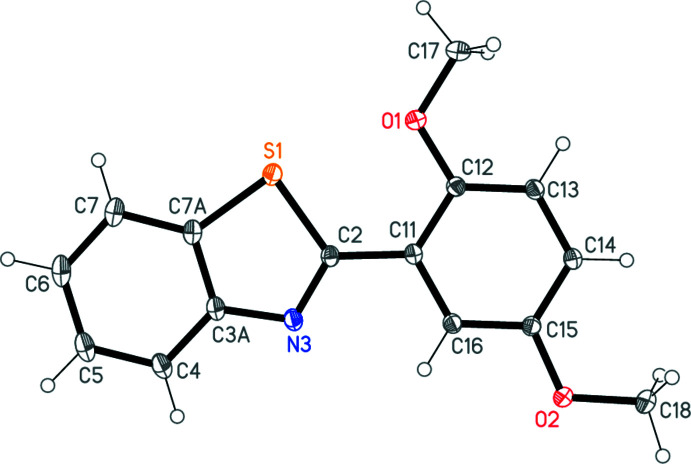
The mol­ecule of **3** in the crystal. Ellipsoids represent 50% probability levels.

The structure of **3** is shown in Fig. 1 ▸. Mol­ecular dimensions may be regarded as normal; a brief selection is presented in Table 1 ▸. Both ring systems are effectively planar (r.m.s. values of 0.01 Å for the benzo­thia­zole and 0.004 Å for the phenyl ring, respectively), with an inter­planar angle of 5.38 (2)°. The approximate coplanarity leads to the short intra­molecular contacts S1⋯O1 = 2.7082 (4) and H16⋯N3 = 2.48 Å; the C16—H16⋯N3 angle is 101°. 
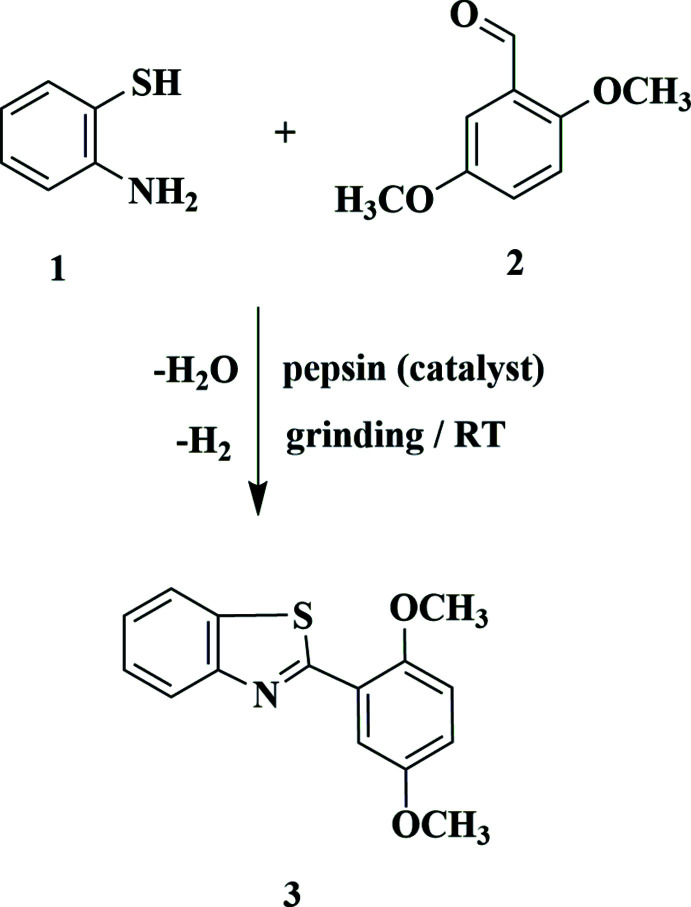


## Supra­molecular features

**Figure 2:**
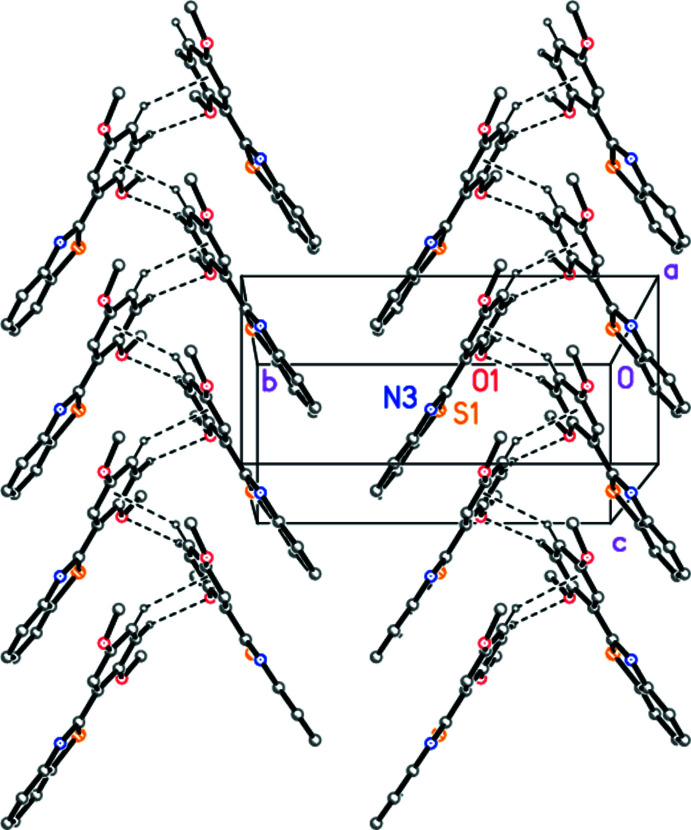
Crystal packing of **3**, viewed perpendicular to (100) in the region *x* ≃ 0.25. Dashed lines indicate ‘weak’ C—H⋯O hydrogen bonds or C—H⋯π contacts. Atom labels correspond to the asymmetric unit.

There are no markedly short inter­molecular contacts. One borderline ‘weak’ C—H⋯O hydrogen bond can be identified (Table 2 ▸), which links mol­ecules *via* the *c-*glide operator *x*, −*y* + 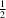 , *z* − 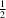 . This is reinforced by a C—H⋯π contact from H14 to the centroid of the phenyl ring (H14⋯*Cg =* 2.67 Å, C14—H14⋯*Cg =* 138°; *Cg* is the centroid of the C11–C16 ring). Additionally, the mol­ecules are linked in pairs, related by *c*-axis translation, in which the benzo­thia­zole ring system of one mol­ecule lies opposite the phenyl ring of the other; the inter­centroid distances are 3.5651 (3) Å for benzo⋯phenyl, and 3.6022 (3) Å for thia­zole⋯phenyl (phenyl operator *x*, *y*, −1 + *z*). The net effect is to form a somewhat flattened herringbone pattern parallel to the *c* axis (Fig. 2 ▸; the π–π inter­actions are not shown explicitly). The contact C18—H18*C*⋯N3 (Table 2 ▸), involving a methyl group, connects the chains in the third dimension *via* the operator −*x* + 1, −*y* + 1, −*z*.

## Database survey

A search of the Cambridge Database (Version 2021.3.0; Groom *et al.*, 2016 ▸) gave four hits for purely organic, neutral species in which one benzo[*d*]thia­zole is bonded at its 2-position to an aromatic C_6_ ring with oxygen substituents at the *ortho* (2-) and *meta* (5-) positions. These were CEFWOB [Yousuf *et al.*, 2012 ▸; 2-hy­droxy, 5-meth­oxy; no S⋯O contact because of an intra­molecular O—H⋯N hydrogen bond; inter­planar angle 1.23 (9)°]; NOYSOM [Wang *et al.*, 2019 ▸; 2,5-dimeth­oxy with an additional 4-(2-pyrid­yl) substituent; two independent mol­ecules; S⋯O = 2.650, 2.715 Å; inter­planar angles of 6.0, 5.5°]; UFAHUF [Chen, 2007 ▸; 2,4,5-trimeth­oxy; S⋯O = 2.671 Å, inter­planar angle of 4.5 (2)°] and WACPUO (Sakai *et al.*, 2016 ▸; 2-hy­droxy, 5-meth­oxy with an additional 3-imidazole substituent; S⋯O = 2.695 Å, inter­planar angle of 1.6°). Where not given in the original publications, these values were calculated using the CCDC program *Mercury* (Macrae *et al.*, 2020 ▸).

## Synthesis and crystallization

A mixture of *o*-amino­thio­phenol **1** (0.01 mol), 2,5-di­meth­oxy­benzaldehyde **2** (0.01 mol) and pepsin (0.05 g) was ground together at room temperature for 10 min. The viscous mixture was poured onto ice–water; the solid that formed was filtered off and recrystallized from ethanol to give pale-yellow crystals of **3** in 97% yield, m.p. 414 K; IR (KBr, cm^−1^): ν_max_ 1581 (C=N); ^1^H NMR (DMSO-*d*_6_): δ = 3.82 (*s*, 3H, OCH_3_), 4.0 (*s*, 3H, OCH_3_), 7.13–7.15 (*m*, 1H, Ar), 7.22 (*d*, 1H, *J* = 8.8 Hz, Ar), 7.41 (*t*, 1H, *J* = 7.6 Hz, Ar), 7.51 (*t*, 1H, J = 7.6 Hz, Ar), 7.96 (*s*, 1H, Ar), 8.07 (*dd*, 2H, *J* = 8.0 Hz, Ar), ^13^C NMR (DMSO-*d*_6_): δ = 56.0, 57.0, 112.5, 114.7, 119.1, 122.1, 122.2, 122.9, 125.4, 126.7, 136.0, 151.9, 153.8, 154.4, 162.3; *m*/*z* = 271 (*M* ^+^, 100%), 238 (61.4%), 185 (27.6%), 136 (79.0%); Analysis: calculated for C_15_H_13_NO_2_S (271.33) C 66.40, H 4.83, N 5.16, S 11.82%; found C 66.58, H 4.65, N 5.39, S 11.68%.

## Refinement

Crystal data, data collection and structure refinement details are summarized in Table 3 ▸. Methyl groups were refined as idealized rigid groups allowed to rotate but not tip (AFIX 137), with C—H = 0.98 Å, H—C—H = 109.5°. Other hydrogen atoms were included using a riding model starting from calculated positions (C—H_aromatic_ = 0.95 Å). The *U*(H) values were fixed at 1.5 or 1.2 × *U*_eq_ of the parent carbon atoms for methyl and aromatic hydrogens, respectively.

## Supplementary Material

Crystal structure: contains datablock(s) I, global. DOI: 10.1107/S2056989022003279/vm2262sup1.cif

Structure factors: contains datablock(s) I. DOI: 10.1107/S2056989022003279/vm2262Isup2.hkl

Supporting information file. DOI: 10.1107/S2056989022003279/vm2262Isup3.cml

CCDC reference: 2161465

Additional supporting information:  crystallographic information; 3D view; checkCIF report

## Figures and Tables

**Table 1:** Selected geometric parameters (Å, °)

S1—C7*A*	1.7327 (6)	N3—C3*A*	1.3820 (7)
S1—C2	1.7642 (5)	C3*A*—C7*A*	1.4082 (8)
C2—N3	1.3064 (7)		
			
C7*A*—S1—C2	89.40 (3)	N3—C3*A*—C7*A*	115.25 (5)
N3—C2—S1	114.72 (4)	C3*A*—C7*A*—S1	109.23 (4)
C2—N3—C3*A*	111.39 (5)		

**Table 2:** Hydrogen-bond geometry (Å, °)

*D*—H⋯*A*	*D*—H	H⋯*A*	*D*⋯*A*	*D*—H⋯*A*
C13—H13⋯O1^i^	0.95	2.65	3.5113 (7)	151
C18—H18*C*⋯N3^ii^	0.98	2.60	3.4867 (8)	151

Symmetry codes: (i) 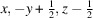 ; (ii) 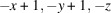 .

**Table 3:** Experimental details

Crystal data	
Chemical formula	C_15_H_13_NO_2_S
*M*_r_	271.32
Crystal system, space group	Monoclinic, *P*2_1_/*c*
Temperature (K)	100
*a*, *b*, *c* (Å)	14.6666 (2), 13.8922 (2), 6.26063 (10)
β (°)	100.1273 (14)
*V* (Å^3^)	1255.74 (3)
*Z*	4
Radiation type	Mo *K*α
μ (mm^−1^)	0.25
Crystal size (mm)	0.22 × 0.22 × 0.15

Data collection	
Diffractometer	XtaLAB Synergy, HyPix
Absorption correction	Multi-scan (*CrysAlis PRO*; Rigaku OD, 2021 ▸)
*T*_min_, *T*_max_	0.927, 1.000
No. of measured, independent and observed [*I* > 2σ(*I*)] reflections	123768, 6759, 6156
*R*_int_	0.023
(sin θ/λ)_max_ (Å^−1^)	0.871

Refinement	
*R*[*F* ^2^ > 2σ(*F* ^2^)], *wR*(*F* ^2^), *S*	0.027, 0.084, 1.04
No. of reflections	6759
No. of parameters	174
H-atom treatment	H-atom parameters constrained
Δρ_max_, Δρ_min_ (e Å^−3^)	0.62, −0.19

Computer programs: *CrysAlis PRO* (Rigaku OD, 2021 ▸), *SHELXT* (Sheldrick, 2015*a* ▸), *SHELXL2018/3* (Sheldrick, 2015*b* ▸) and *XP* (Siemens, 1994 ▸).

## supplementary crystallographic information

### Crystal data

**Table:**

C_15_H_13_NO_2_S	*F*(000) = 568
*M**_r_* = 271.32	*D*_x_ = 1.435 Mg m^−^^3^
Monoclinic, *P*2_1_/*c*	Mo *K*α radiation, λ = 0.71073 Å
*a* = 14.6666 (2) Å	Cell parameters from 88513 reflections
*b* = 13.8922 (2) Å	θ = 2.8–38.4°
*c* = 6.26063 (10) Å	µ = 0.25 mm^−^^1^
β = 100.1273 (14)°	*T* = 100 K
*V* = 1255.74 (3) Å^3^	Block, colourless
*Z* = 4	0.22 × 0.22 × 0.15 mm

### Data collection

**Table:**

XtaLAB Synergy, HyPix diffractometer	6759 independent reflections
Radiation source: micro-focus sealed X-ray tube	6156 reflections with *I* > 2σ(*I*)
Detector resolution: 10.0000 pixels mm^-1^	*R*_int_ = 0.023
ω scans	θ_max_ = 38.3°, θ_min_ = 2.0°
Absorption correction: multi-scan (CrysAlisPro; Rigaku OD, 2021)	*h* = −24→25
*T*_min_ = 0.927, *T*_max_ = 1.000	*k* = −23→23
123768 measured reflections	*l* = −10→10

### Refinement

**Table:**

Refinement on *F*^2^	Primary atom site location: dual
Least-squares matrix: full	Hydrogen site location: inferred from neighbouring sites
*R*[*F*^2^ > 2σ(*F*^2^)] = 0.027	H-atom parameters constrained
*wR*(*F*^2^) = 0.084	*w* = 1/[σ^2^(*F*_o_^2^) + (0.0489*P*)^2^ + 0.2455*P*] where *P* = (*F*_o_^2^ + 2*F*_c_^2^)/3
*S* = 1.03	(Δ/σ)_max_ = 0.001
6759 reflections	Δρ_max_ = 0.62 e Å^−^^3^
174 parameters	Δρ_min_ = −0.19 e Å^−^^3^
0 restraints	

### Special details

**Table:**

Geometry. All esds (except the esd in the dihedral angle between two l.s. planes) are estimated using the full covariance matrix. The cell esds are taken into account individually in the estimation of esds in distances, angles and torsion angles; correlations between esds in cell parameters are only used when they are defined by crystal symmetry. An approximate (isotropic) treatment of cell esds is used for estimating esds involving l.s. planes. Least-squares planes (x,y,z in crystal coordinates) and deviations from them (* indicates atom used to define plane) - 0.2606 (0.0025) x + 11.3686 (0.0009) y - 3.5208 (0.0006) z = 4.3561 (0.0009) * -0.0113 (0.0003) S1 * 0.0122 (0.0004) C2 * 0.0073 (0.0004) N3 * -0.0022 (0.0005) C3A * -0.0178 (0.0004) C4 * 0.0029 (0.0005) C5 * 0.0151 (0.0005) C6 * -0.0033 (0.0005) C7 * -0.0029 (0.0005) C7A Rms deviation of fitted atoms = 0.0101 - 1.6150 (0.0031) x + 11.4261 (0.0017) y - 3.3180 (0.0011) z = 4.1087 (0.0012) Angle to previous plane (with approximate esd) = 5.381 ( 0.023 ) * -0.0035 (0.0003) C11 * 0.0058 (0.0004) C12 * -0.0025 (0.0004) C13 * -0.0031 (0.0004) C14 * 0.0054 (0.0004) C15 * -0.0020 (0.0004) C16 -0.0003 (0.0008) O1 0.0801 (0.0011) C17 0.0285 (0.0008) O2 0.1636 (0.0010) C18 Rms deviation of fitted atoms = 0.0040

### Fractional atomic coordinates and isotropic or equivalent isotropic displacement parameters (Å^2^)

**Table:**

	*x*	*y*	*z*	*U*_iso_*/*U*_eq_	
S1	0.13465 (2)	0.49165 (2)	0.34350 (2)	0.01451 (4)	
C2	0.24674 (4)	0.47726 (4)	0.28209 (8)	0.01188 (8)	
N3	0.31247 (3)	0.52002 (3)	0.41668 (7)	0.01347 (7)	
C3A	0.27752 (4)	0.56826 (4)	0.57771 (8)	0.01360 (8)	
C4	0.33134 (4)	0.62033 (4)	0.74630 (9)	0.01695 (9)	
H4	0.396755	0.623486	0.758537	0.020*	
C5	0.28692 (5)	0.66707 (4)	0.89466 (9)	0.01963 (10)	
H5	0.322334	0.703577	1.008219	0.024*	
C6	0.19042 (5)	0.66133 (5)	0.87976 (10)	0.02095 (11)	
H6	0.161650	0.694472	0.982862	0.025*	
C7	0.13610 (5)	0.60805 (4)	0.71696 (10)	0.01924 (10)	
H7	0.071002	0.603042	0.709129	0.023*	
C7A	0.18087 (4)	0.56196 (4)	0.56475 (9)	0.01468 (8)	
C11	0.26660 (3)	0.42388 (3)	0.09270 (8)	0.01131 (7)	
C12	0.19879 (3)	0.37320 (4)	−0.05164 (8)	0.01179 (8)	
C13	0.22303 (4)	0.32478 (4)	−0.22766 (8)	0.01295 (8)	
H13	0.177114	0.290066	−0.323243	0.016*	
C14	0.31402 (4)	0.32648 (4)	−0.26592 (8)	0.01283 (8)	
H14	0.329903	0.293120	−0.386674	0.015*	
C15	0.38132 (3)	0.37743 (4)	−0.12577 (8)	0.01215 (8)	
C16	0.35765 (4)	0.42522 (4)	0.05254 (8)	0.01240 (8)	
H16	0.404004	0.459248	0.148423	0.015*	
O1	0.11061 (3)	0.37347 (3)	−0.00594 (7)	0.01587 (7)	
C17	0.04056 (4)	0.32676 (5)	−0.15693 (10)	0.01998 (10)	
H17A	0.036645	0.356648	−0.300050	0.030*	
H17B	−0.019153	0.333071	−0.108453	0.030*	
H17C	0.056005	0.258430	−0.166186	0.030*	
O2	0.47191 (3)	0.38509 (3)	−0.15047 (7)	0.01691 (8)	
C18	0.49545 (4)	0.34434 (5)	−0.34295 (10)	0.01952 (10)	
H18A	0.454902	0.371139	−0.470203	0.029*	
H18B	0.487554	0.274329	−0.340465	0.029*	
H18C	0.560067	0.359596	−0.350301	0.029*	

### Atomic displacement parameters (Å^2^)

**Table:**

	*U*^11^	*U*^22^	*U*^33^	*U*^12^	*U*^13^	*U*^23^
S1	0.01415 (6)	0.01602 (6)	0.01373 (6)	0.00148 (4)	0.00352 (4)	−0.00175 (4)
C2	0.01436 (18)	0.01083 (17)	0.01076 (17)	0.00014 (14)	0.00307 (14)	0.00011 (13)
N3	0.01641 (18)	0.01302 (17)	0.01139 (16)	−0.00157 (13)	0.00354 (13)	−0.00175 (13)
C3A	0.0193 (2)	0.01086 (18)	0.01120 (17)	−0.00042 (15)	0.00426 (15)	−0.00038 (14)
C4	0.0236 (2)	0.0142 (2)	0.01336 (19)	−0.00312 (17)	0.00403 (17)	−0.00254 (15)
C5	0.0314 (3)	0.0140 (2)	0.0142 (2)	−0.00201 (19)	0.00606 (19)	−0.00317 (16)
C6	0.0321 (3)	0.0161 (2)	0.0167 (2)	0.0025 (2)	0.0097 (2)	−0.00306 (17)
C7	0.0239 (2)	0.0180 (2)	0.0176 (2)	0.00356 (19)	0.00846 (19)	−0.00219 (18)
C7A	0.0190 (2)	0.01267 (19)	0.01312 (18)	0.00175 (15)	0.00489 (15)	−0.00059 (15)
C11	0.01340 (17)	0.01037 (17)	0.01029 (16)	0.00010 (13)	0.00241 (13)	−0.00034 (13)
C12	0.01206 (17)	0.01134 (18)	0.01187 (17)	0.00064 (13)	0.00182 (14)	−0.00025 (13)
C13	0.01318 (18)	0.01267 (18)	0.01268 (18)	0.00015 (14)	0.00138 (14)	−0.00222 (14)
C14	0.01418 (18)	0.01223 (18)	0.01217 (18)	0.00012 (14)	0.00257 (14)	−0.00183 (14)
C15	0.01276 (18)	0.01177 (18)	0.01232 (17)	−0.00073 (14)	0.00333 (14)	−0.00073 (14)
C16	0.01365 (18)	0.01212 (18)	0.01161 (17)	−0.00128 (14)	0.00273 (14)	−0.00118 (14)
O1	0.01170 (15)	0.01927 (18)	0.01672 (16)	−0.00048 (12)	0.00270 (12)	−0.00411 (13)
C17	0.01240 (19)	0.0272 (3)	0.0192 (2)	−0.00078 (18)	−0.00011 (17)	−0.0035 (2)
O2	0.01379 (16)	0.02132 (19)	0.01681 (17)	−0.00358 (13)	0.00599 (13)	−0.00600 (14)
C18	0.0175 (2)	0.0238 (3)	0.0190 (2)	−0.00194 (19)	0.00813 (18)	−0.00580 (19)

### Geometric parameters (Å, º)

**Table:**

S1—C7A	1.7327 (6)	C15—O2	1.3686 (6)
S1—C2	1.7642 (5)	C15—C16	1.3941 (7)
C2—N3	1.3064 (7)	O1—C17	1.4243 (7)
C2—C11	1.4704 (7)	O2—C18	1.4276 (7)
N3—C3A	1.3820 (7)	C4—H4	0.9500
C3A—C4	1.4027 (8)	C5—H5	0.9500
C3A—C7A	1.4082 (8)	C6—H6	0.9500
C4—C5	1.3862 (8)	C7—H7	0.9500
C5—C6	1.4043 (10)	C13—H13	0.9500
C6—C7	1.3912 (9)	C14—H14	0.9500
C7—C7A	1.4032 (8)	C16—H16	0.9500
C11—C16	1.4017 (7)	C17—H17A	0.9800
C11—C12	1.4091 (7)	C17—H17B	0.9800
C12—O1	1.3728 (6)	C17—H17C	0.9800
C12—C13	1.3896 (7)	C18—H18A	0.9800
C13—C14	1.3969 (7)	C18—H18B	0.9800
C14—C15	1.3930 (7)	C18—H18C	0.9800
			
C7A—S1—C2	89.40 (3)	C15—O2—C18	116.63 (4)
N3—C2—C11	121.40 (5)	C5—C4—H4	120.7
N3—C2—S1	114.72 (4)	C3A—C4—H4	120.7
C11—C2—S1	123.86 (4)	C4—C5—H5	119.5
C2—N3—C3A	111.39 (5)	C6—C5—H5	119.5
N3—C3A—C4	124.55 (5)	C7—C6—H6	119.4
N3—C3A—C7A	115.25 (5)	C5—C6—H6	119.4
C4—C3A—C7A	120.20 (5)	C6—C7—H7	121.2
C5—C4—C3A	118.50 (6)	C7A—C7—H7	121.2
C4—C5—C6	121.05 (6)	C12—C13—H13	119.6
C7—C6—C5	121.30 (5)	C14—C13—H13	119.6
C6—C7—C7A	117.66 (6)	C15—C14—H14	120.2
C7—C7A—C3A	121.26 (5)	C13—C14—H14	120.2
C7—C7A—S1	129.51 (5)	C15—C16—H16	119.5
C3A—C7A—S1	109.23 (4)	C11—C16—H16	119.5
C16—C11—C12	118.61 (4)	O1—C17—H17A	109.5
C16—C11—C2	117.91 (4)	O1—C17—H17B	109.5
C12—C11—C2	123.47 (4)	H17A—C17—H17B	109.5
O1—C12—C13	123.26 (5)	O1—C17—H17C	109.5
O1—C12—C11	116.71 (4)	H17A—C17—H17C	109.5
C13—C12—C11	120.02 (5)	H17B—C17—H17C	109.5
C12—C13—C14	120.88 (5)	O2—C18—H18A	109.5
C15—C14—C13	119.54 (5)	O2—C18—H18B	109.5
O2—C15—C14	124.24 (5)	H18A—C18—H18B	109.5
O2—C15—C16	115.90 (4)	O2—C18—H18C	109.5
C14—C15—C16	119.85 (5)	H18A—C18—H18C	109.5
C15—C16—C11	121.10 (5)	H18B—C18—H18C	109.5
C12—O1—C17	117.16 (4)		
			
C7A—S1—C2—N3	0.85 (4)	S1—C2—C11—C16	174.46 (4)
C7A—S1—C2—C11	−177.67 (4)	N3—C2—C11—C12	177.18 (5)
C11—C2—N3—C3A	177.97 (4)	S1—C2—C11—C12	−4.38 (7)
S1—C2—N3—C3A	−0.60 (6)	C16—C11—C12—O1	179.80 (5)
C2—N3—C3A—C4	179.57 (5)	C2—C11—C12—O1	−1.36 (7)
C2—N3—C3A—C7A	−0.08 (7)	C16—C11—C12—C13	0.89 (7)
N3—C3A—C4—C5	178.64 (5)	C2—C11—C12—C13	179.73 (5)
C7A—C3A—C4—C5	−1.73 (8)	O1—C12—C13—C14	−179.65 (5)
C3A—C4—C5—C6	1.12 (9)	C11—C12—C13—C14	−0.81 (8)
C4—C5—C6—C7	0.50 (10)	C12—C13—C14—C15	−0.03 (8)
C5—C6—C7—C7A	−1.46 (9)	C13—C14—C15—O2	−178.84 (5)
C6—C7—C7A—C3A	0.83 (9)	C13—C14—C15—C16	0.78 (8)
C6—C7—C7A—S1	−179.51 (5)	O2—C15—C16—C11	178.96 (5)
N3—C3A—C7A—C7	−179.57 (5)	C14—C15—C16—C11	−0.69 (8)
C4—C3A—C7A—C7	0.76 (8)	C12—C11—C16—C15	−0.14 (7)
N3—C3A—C7A—S1	0.70 (6)	C2—C11—C16—C15	−179.05 (5)
C4—C3A—C7A—S1	−178.96 (4)	C13—C12—O1—C17	−4.35 (8)
C2—S1—C7A—C7	179.48 (6)	C11—C12—O1—C17	176.78 (5)
C2—S1—C7A—C3A	−0.82 (4)	C14—C15—O2—C18	5.35 (8)
N3—C2—C11—C16	−3.97 (7)	C16—C15—O2—C18	−174.28 (5)

### Hydrogen-bond geometry (Å, º)

**Table:**

*D*—H···*A*	*D*—H	H···*A*	*D*···*A*	*D*—H···*A*
C13—H13···O1^i^	0.95	2.65	3.5113 (7)	151
C18—H18*C*···N3^ii^	0.98	2.60	3.4867 (8)	151

Symmetry codes: (i) *x*, −*y*+1/2, *z*−1/2; (ii) −*x*+1, −*y*+1, −*z*.
